# Modulating the Thermoresponse of Polymer-Protein Conjugates with Hydrogels for Controlled Release

**DOI:** 10.3390/polym13162772

**Published:** 2021-08-18

**Authors:** Vincent Huynh, Natalie Ifraimov, Ryan G. Wylie

**Affiliations:** 1 Department of Chemistry and Chemical Biology, McMaster University, Hamilton, ON L8S 4M1, Canada; huynhv2@mcmaster.ca; 2 School of Biomedical Engineering, McMaster University, Hamilton, ON L8S 4M1, Canada; ifraimon@mcmaster.ca

**Keywords:** polymer-protein conjugates, thermoresponsive, controlled release, temperature-sensitive polymers, hydrogels

## Abstract

Sustained release is being explored to increase plasma and tissue residence times of polymer-protein therapeutics for improved efficacy. Recently, poly(oligo(ethylene glycol) methyl ether methacrylate) (PEGMA) polymers have been established as potential PEG alternatives to further decrease immunogenicity and introduce responsive or sieving properties. We developed a drug delivery system that locally depresses the lower critical solution temperature (LCST) of PEGMA-protein conjugates within zwitterionic hydrogels for controlled release. Inside the hydrogel the conjugates partially aggregate through PEGMA-PEGMA chain interactions to limit their release rates, whereas conjugates outside of the hydrogel are completely solubilized. Release can therefore be tuned by altering hydrogel components and the PEGMA’s temperature sensitivity without the need for traditional controlled release mechanisms such as particle encapsulation or affinity interactions. Combining local LCST depression technology and degradable zwitterionic hydrogels, complete release of the conjugate was achieved over 13 days.

## 1. Introduction

Polymer conjugation to protein therapeutics is a common technique to enhance therapeutic efficacy by minimizing immunogenicity and increasing plasma and tissue residence times due to slower clearance rates [1,2]. Conjugation of poly(ethylene) glycol (PEG) is becoming a routine method to synthesize polymer-protein conjugates, with several used clinically [2,3]. To further improve the efficacy of polymer-protein conjugates, alternatives to PEG are being explored to maintain therapeutic effects while further minimizing antigenic or immunogenic effects [1,4]. PEG alternatives include poly(zwitterions), polysaccharides, poly(oligo(ethylene glycol) methyl ether methacrylate) (PEGMA), peptides, and many more [1,5].

PEGMA is an attractive polymer for protein conjugation due to low antigenicity, ease of polymerization and successful demonstrations in the production of polymer-protein conjugates [6,7,8]. For example, PEGMA conjugation to a GLP-1 agonist (exenatide) extended its plasma half-life, which enhanced and prolonged glucose suppression [7] Furthermore, PEGMA with side chains of only 2–3 ethylene glycol repeat units do not bind to anti-PEGs antibodies, potentially reducing immune recognition of the polymer [6]. PEGMA conjugation to proteins has also been shown to display molecular sieving effects, providing an additional strategy for increasing therapeutic half-life and decreasing immune recognition [9,10]. Therefore, PEGMA is well established as a potential alternative to PEG and warrants further exploration.

Despite the greater plasma and tissue half-lives provided upon polymer conjugation, repeated dosing of polymer-protein conjugates are often required to maintain therapeutic drug concentrations at disease sites. This has prompted research into stimuli responsive drug depots that slowly release polymer-protein conjugates to reduce the frequency of administration [11]. For example, temperature responsive elastin like peptides have been conjugated to protein drugs such as exenatide to form a sparingly soluble drug depot upon injection due to a temperature-sensitive phase transition [12,13]. The aggregated polymer-protein conjugate is then slowly released from the drug depot upon dissolution of the conjugate through phase transitions at the surface. This strategy resulted in prolonged glucose suppression in a diabetic mouse model with a single injection. In a similar manner, temperature-sensitive phase transitioning PEGMA conjugates can be used for controlled released applications.

**Figure 1:**
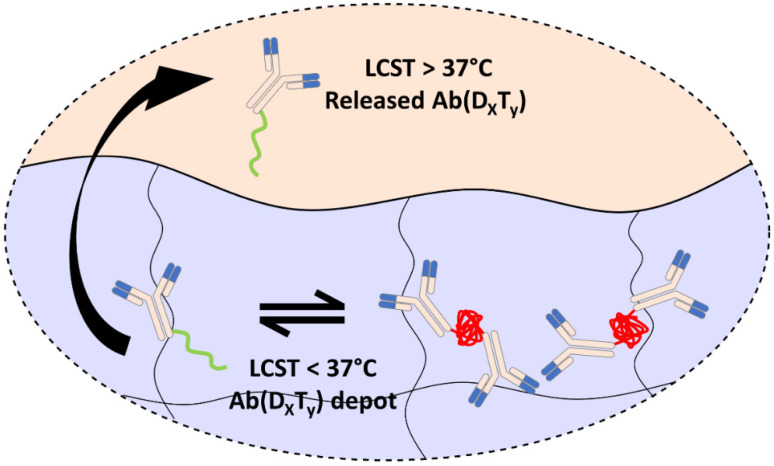
Local LCST depression of PEGMA-antibody conjugates (Ab(D_x_T_y_)) within a zwitterionic hydrogel for the controlled release of solubilized conjugates. Within the hydrogel, the Ab(D_x_T_y_) aggregates form due to hydrogel promoted LCST depression and slowly dissolve for controlled release. PEGMA copolymers of diethylene glycol methyl ether methacrylate (D) and triethylene glycol methyl ether methacrylate (T) P(D_x_T_y_) were synthesized to achieve temperature-sensitive polymers with suitable and hydrogel promoted phase transitions for Ab(D_x_T_y_). The conjugates were designed to undergo phase transitions within but not outside of the hydrogel.

To further enhance the controlled delivery of polymer-protein conjugates, we have developed an injectable hydrogel delivery vehicle that decreases the lower critical solution temperature (LCST) of encapsulated PEGMA-protein conjugates. The conjugates can therefore be designed to phase transition at body temperature within but not outside of the hydrogel, providing new a mechanism for the controlled release of PEGMA-protein conjugates without the need for traditional controlled release mechanisms such as particle formulations or hydrogel binding interactions. Therefore, controlled release can simply be achieved by locally injecting therapeutic PEGMA-protein conjugates with hydrogels, simplifying formulations for potential clinical applications. By exploiting the influence of Hofmeister salt series in decreasing the LCST of PEGMA, we developed a zwitterionic hydrogel to influence PEGMA’s LCST [14]. The zwitterionic hydrogel changes the local electrochemical environment, thereby lowering the LCST of an encapsulated PEGMA-protein conjugate, yielding a drug depot where release is governed by the dissolution of the formed aggregates and the hydrogel degradation rate. To ensure the PEGMA-protein conjugates form an aggregate depot within the hydrogel but not after their release from the hydrogel, the conjugate was designed to have an LCST above 37 °C in a buffered solution and below 37 °C in the hydrogel (Figure 1).

## 2. Materials and Methods

### 2.1. Materials 

4-Cyano-4-(phenylcarbonothioylthio)pentanoic acid (CTA1), 4,4′-azobis(4-cyanovaleric acid) (ACVA), triethylamine, 2-(dodecylthiocarbonothioylthio)-2-methylpropionic acid 3-azido-1-propanol ester (CTA2), triethylene glycol methyl ether methacrylate, diethylene glycol methyl ether methacrylate, poly(ethylene glycol) methyl ether methacrylate (M_n_ 500) (P(EG_9_)MA), 2-methacroloyloxyethyl phosphorylcholine (MPC), [2-(methacryloyloxy)ethyl]dimethyl-(3-sulfopropyl)ammonium hydroxide (SB), were purchased from Sigma Aldrich (Oakville, ON, Canada). N-(3-aminopropyl)methacrylamide hydrochloride (APMA) was purchased from Polysciences, Inc. (Warrington, PA, USA). Dibenzocyclooctyne-PEG_4_-NHS (DBCO-PEG_4_-NHS) was purchased from Click Chemistry Tools (Scottsdale, AZ, USA). 3-(3-dimethylaminopropyl)-1-ethyl-carbodiimide hydrochloride (EDC-HCl) was obtained from Chem-Impex International Inc. (Wood Dale, IL, USA) Alexa Fluor 647 NHS ester, Alexa Fluor 488 NHS Ester, Fetal bovine serum (FBS)DMEM, Alamar blue was purchased from Thermo Fisher Scientific (Burlington, ON, Canada). O-(6-azidohexyl)-O’-succinimidyl carbonate (NHS-azide), O-[1-(4-chlorophenylsulfonyl)-7-azido-2-heptyl]-O′-succinimidylcarbonate (NHS-AzCl) and carboxybetaine monomer were synthesized as previously described [15,16,17].

### 2.2. Methods

Poly(carboxybetaine) (pCB) hydrogel synthesis. pCB hydrogels were synthesized in a similar manner as previously described [15,17,18]. Copolymers of pCB-APMA, pCB-Azide (pCB-Az), pCB-AzCl and pCB-DBCO were synthesized as described below. Hydrogels were fabricated by mixing pCB-DBCO and pCB-Az at desired concentration. 

pCB-APMA synthesis. CB monomer was synthesized according to previous protocols. CB monomer (1 g) and APMA-HCl (20 mg) was dissolved in 2.893 mL 1M sodium acetate buffer. Separately, CTA1 (5.5 mg) and ACVA (1.1) was dissolved in 579 µL dioxane. The reagents were added together resulting in 5:1 buffer: dioxane. The pH of the solution was then adjusted to pH 3–4 and was then placed in a Schlenk flask, subject to 3 cycles of freeze-pump-thaw and backfilled with N_2_. The vessel was then submerged in an oil bath at 70 °C and reacted for 2 d. The polymerization was terminated by exposure to air and the solution was dialyzed against water (pH 3–4) for 3 d (MWCO 12–14k) and lyophilized yielding a pink powder. Polymer molecular weight and dispersity was determined with gel permeation chromatography using PEG standards. 

pCB-Az, pCB-AzCl and pCB-DBCO synthesis. pCB-APMA random copolymers were synthesized according to previous procedures. pCB-APMA polymers were then functionalized with azide or DBCO moieties. We dissolved 500 mg of pCB-APMA in 5 mL of dry MeOH with 50 μL (0.50 mmol) of triethylamine. Subsequently, NHS-azide (14 mg, 0.05 mmol), NHS-DBCO (20 mg, 0.05 mmol) or NHS-AzCl (23 mg, 0.05 mmol) was added and reacted overnight under N_2_ at room temperature. Polymers were then precipitated with 45 mL diethyl ether, dried and dissolved in 10–15 mL of deionized water. The aqueous solutions were then extracted twice with dichloromethane (DCM) and the aqueous layer was then dialyzed against water for 1 d (MWCO 12–14k). The polymers were then lyophilized yielding a white or pink powder. Polymer composition was determined by ^1^H NMR upon integrating the methylene peak in the backbone with unique resonances for pCB-Az, pCB-AzCl (hydrogen adjacent to the carbamate bond) and pCB-DBCO (aromatic DBCO moieties; Figure S5).

P(D_x_T_y_) Polymer Synthesis. We mixed 0.5 g of triethylene glycol methyl ether methacrylate (T) was mixed with di(ethylene glycol) methyl ether methacrylate (D) to yield PEGMA copolymers of varying D and T mole fractions. For T homopolymer and copolymers of D_10_T_90_, D_20_T_80_, D_30_T_70_, D_40_T_60_ and D_50_T_50_, 0, 45, 101, 174, 270 and 405 mg for D was added, respectively, to the 0.5 mg solution of T along with a 3 mL dioxane solution of CTA2 (8.2 mg) and initiator (1.2 mg). The reaction mixture was then transferred to a Schlenk flask and underwent three freeze-pump-thaw cycles, followed by a nitrogen backfill. The reaction was then submerged in a 70 °C oil bath and reacted for 16 h. The reaction was then dialyzed against water (MWCO 12–14k) for 3 d. The product was then lyophilized yielding a yellow oil.

P(EG_9_)MA synthesis. Poly(ethylene glycol) methyl ether methacrylate (M_n_ 500) (1 g), CTA1 (5.6 mg) and ACVA (1.1 mg) was dissolved in 993 µL dioxane. The reaction mixture was then transferred to a Schlenk flask and underwent three freeze-pump-thaw cycles, followed by a nitrogen backfill. The reaction was then submerged in a 70 °C oil bath and reacted for 16 h. The reaction was then dialyzed against water at pH 3–4 (MWCO 12–14k) for 3 d and then lyophilized yielding a pink oil.

pMPC synthesis. MPC monomer (0.75 mg), CTA1 (4.2) and ACVA (1 mg) was dissolved in 1.787 mL of methanol. The reaction mixture was then sparged with nitrogen for 45 min, immersed in a 70 °C oil bath and reacted for 16 h. The polymer was then precipitated in acetone and dried overnight in the vacuum oven. The polymer was then dissolved in water and dialyzed against water at pH 3–4 (MWCO 12–14k) for 1 day. The product was then lyophilized, yielding a pink powder.

pSB synthesis. SB monomer (1 g), CTA1 (5.6 mg) and ACVA (1.1 mg) was dissolved in 2.380 mL 0.5 M NaCl. The reaction mixture was then transferred to a Schlenk flask and underwent three freeze-pump-thaw cycles, followed by a nitrogen backfill. The reaction was then submerged in a 70 °C oil bath and reacted for 16h. The reaction was then dialyzed against pH 3–4 water (MWCO 12–14k) for 3 d. The product was then lyophilized yielding a pink powder.

pCB synthesis. CB monomer (1 g), CTA1 (9.4 mg) and ACVA (1.9 mg) was dissolved in 3.375 mL 1 M acetate buffer (pH 5.2), the pH was the adjusted to pH 3–4. The reaction mixture was then transferred to a Schlenk flask and underwent three freeze-pump-thaw cycles and was backfilled with nitrogen. The reaction was then submerged in a 70 °C oil bath and reacted for 48 h. The reaction was then dialyzed against water at pH 3–4 (MWCO 12–14k) for 3 d. The product was then lyophilized yielding a pink powder.

Polymer LCST determination. D_x_T_y_ polymers were dissolved in PBS (10 mg mL^−1^) overnight at 4 °C. The polymers were then mixed with a solution of the modulating polymer (P(EG_9_)MA, pMPC, pCB, pSB, pCB-APMA, pCB-Az, pCB-DBCO, PBS) resulting in a final concentration of 50 mg mL^−1^ of the modulating polymer and 5 mg mL^−1^ of the D_x_T_y_ polymer in PBS. The sample was then transferred to a 1 mL quartz cuvette and the optical density at 550 nm (OD550) was measured using an Agilent UV Vis NIR spectrophotometer equipped with a Peltier accessory. Similarly, D_x_T_y_ polymers were mixed with either pCB-DBCO and pCB-Az resulting in a final concentration of 100 mg mL^−1^ of pCB-DBCO/pCB-Az and 5 mg mL^−1^ D_x_T_y_ polymer. The two mixtures were then combined in a 1 mL quartz cuvette and gelled for 30 min at room temperature and the optical density at 550 nm (OD550) was measured using an Agilent UV Vis NIR spectrophotometer equipped with a Peltier accessory. The sample was heated from 5 °C to 75 °C at a rate of 3 °C per minute and cooled from 75 °C to 5 °C at 3 °C per minute. OD550 measurements were taken every 0.5 °C.

Ab-DBCO and Ab-DBCO-488 synthesis. We added 33 μL of 10 mg mL^−1^ DBCO-PEG_4_-NHS dissolved in DMSO to 1 mL of Avastin (10 mg mL^−1^; Ab) in borax buffer (0.1 M borax, 0.15 M NaCl, pH 8.75) and reacted overnight at room temperature. Avastin-DBCO (Ab-DBCO) was purified by size exclusion chromatography using a GE Healthcare Superdex S200 column. Fractions were collected and concentrated using an Amicon Spin concentrator (MWCO 30k). DBCO conjugation was confirmed and quantified by UV Vis. Number of DBCO’s conjugated to Av was determined using previously described methods [19].

Ab-DBCO-488 was then synthesized. Briefly, 2 μL of Alexa Fluor 488 NHS ester (10 mg mL^−1^) was added to 300 μL of Ab-DBCO (5 mg mL^−1^) in PBS and reacted at room temperature for 3 h in the dark. The reaction was then dialyzed against PBS (MWCO 12–14k) at 4 °C in the dark for 3 d. Ab-488 was synthesized as a control. Briefly, 2 μL of Alexa Fluor 488 was added to 500 μL of Ab in PBS (10 mg mL^−1^) and incubated at room temperature for 3h in the dark. The solution was subsequently dialyzed against PBS (MWCO 12–14k) at 4 °C in the dark for 3 d.

Ab(D_x_T_y_) synthesis and validation. We added 125 μL of D_X_T_y_ (25 mg mL^−1^) dissolved in PBS to 125 μL of Av-DBCO (5 mg mL^−1^) in PBS and reacted overnight at room temperature with gentle agitation. Ab(D_x_T_y_) was purified by size exclusion chromatography, using a GE Healthcare Superdex S200 column. Fractions were collected and concentrated with an Amicon Spin concentrator (MWCO 100k).

To ensure complete reaction of DBCO’s conjugated Avastin, 4 μL of Ab-DBCO (5 mg mL^−1^) in PBS was first added to 4 μL of D_x_T_y_ (25 mg mL^−1^) dissolved in PBS and reacted overnight at room temperature. We then added 2 μL of N_3_-Cy5 dye in water and reacted at room temperature overnight in the dark. Samples were then loaded onto an SDS PAGE and run at 120 V for 45 min. The gel was imaged using a Biorad Chemidoc using the Cy5 filter and processed using ImageLab software. The appearance of fluorescent bands would indicate the presence of unreacted DBCO’s on Av.

Hydrogel degradation. On the bottom of pre-weighted 2 mL Eppendorf tubes, 10 wt% AzCl hydrogels were formed. Hydrogels were then allowed to gel at room temperature for 30 min to ensure complete gelation. We carefully pipetted 1 mL of PBS over top the gels, which were then incubated at 37 °C. At specific time intervals, PBS was removed, and the surface of the gels were dabbed with a kimwipe. Wet gel weights were then measured and plotted against time.

Ab(D_x_T_y_) LCST determination. Ab(D_x_T_y_) conjugates (0.5 mg mL^−1^) were transferred to a 100 μL quartz cuvette and OD550 was measured using an Agilent UV Vis NIR spectrophotometer equipped with a Peltier accessory. Samples were heated from 15 to 60 °C at a rate of 0.5 °C per minute and then cooled from 60 to 15 °C at 0.5 °C per minute. OD550 readings were taken every 0.5 °C.

Controlled release experiments of Ab(D_x_T_y_) from pCB hydrogels. Ab(D_x_T_y_)-488 in PBS (2 mg mL^−1^) was incubated without or with D_x_T_y_ polymers (0, 2.5 or 5 mgmL^−1^) dissolved in PBS (40 mg mL^−1^) overnight at 4 °C in the dark. The solution was then mixed with pCB-DBCO and pCB-Az forming 60 μL gels on the bottom of a black 96-well plate resulting in 10 wt% pCB hydrogels containing 0, 2.5 or 5 mg mL^−1^ D_x_T_y_ polymers and 0.25 mg mL^−1^ Ab-DBCO-488. Gels were incubated at 37 °C for 1 h to ensure gelation. We pipetted 200 μL of warm (37 °C) PBS on top of the gels. Supernatants were taken at specific time intervals and replenished with warm PBS. Supernatant fluorescence was read using a Biotek Cytation 5 plate reader (Ex. 495 nm, Em. 519 nm) and amounts of released Ab(D_x_T_y_) were determined using a calibration curve.

Cytotoxicity assay. Human lung fibroblasts (HLF) (10,000 cells) were seeded onto a clear flat bottom 96-well plate with growth medium (DMEM, 10% FBS). D_x_T_y_ polymers dissolved in PBS (1 mg mL^−1^) were then added to each well. The cells were incubated at 37 °C 5% CO_2_ for 24h. Alamar Blue (Invitrogen), was then added to each well and further incubated at 37 °C 5% CO_2_ for 1 h. Fluorescence was then measured using a Cytation 5 plate reader (ex. 560 nm, em. 590 nm). Fluorescence readings were normalized to a control where PBS was added.

## 3. Results and Discussion

### 3.1. Selection of Polymer Components for Hydrogel and Antibody Conjugates

**Figure 2:**
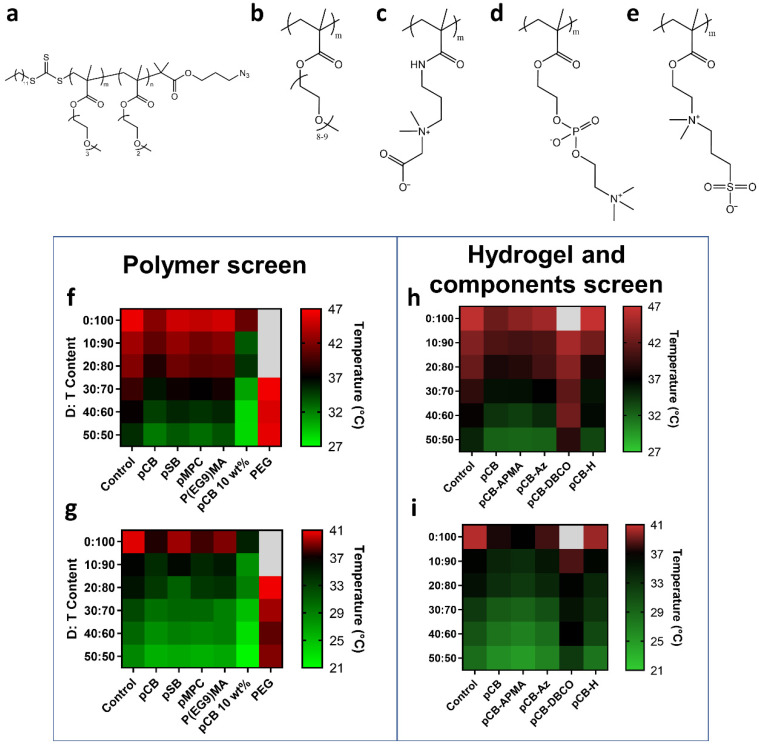
LCST screen of P(D_x_T_y_) copolymers with varying D:T content in the presence of hydrophilic polymers, hydrogel components and encapsulated in pCB hydrogels. Structures of polymers used in the study: (**a**) P(D_x_T_y_), (**b**) P(EG_9_)MA, (**c**) pCB, (**d**) pMPC, (**e**) pSB. (**f**,**h**) Heat map of LCST transition temperatures (at 50% of maximum turbidity of heating curves) of different PEGMA copolymer compositions (10 mg/mL) in combination with modulating polymers (50 mg/mL unless specified). (**g**,**i**) Heat map of LCST cooling transition temperatures (at 50% of maximum turbidity of cooling curves) of different PEGMA polymer compositions (10 mg/mL) in combination with modulating polymers (50 mg/mL unless specified). Colored temperature scales are presented with 37 °C set as black.

To identify suitable polymers for antibody conjugates and injectable hydrogels for LCST modulating delivery vehicles, we first synthesized azide terminated PEGMA random copolymers with various D:T ratios with MWs of 26–40k (P(D_x_T_y_); Figure 2a, Figure S1, Table S1), and thus LCSTs, for conjugation to antibodies. The LCST, midpoint (50%) of turbidity curves, of 0.5 wt% P(D_x_T_y_) solutions in PBS was determined in the presence of different hydrophilic polymers that are either zwitterionic (pCB, pSB or pMPC) or PEGMA with greater hydrophilicity (P(EG_9_)MA) than P(D_x_T_y_) copolymers (Figure S2). As expected, hydrophilic polymers decreased the LCST of all P(D_x_T_y_) copolymers, with pCB resulting in the greatest (~3.2 °C) shift (Figure 2f, Figures S3 and S4), most likely due to pCB’s greater ability to form a water shell under physiological conditions and known ability to promote hydrophobic interactions. This is similar to previous reports where the hydration state of pCB enhanced hydrophobic interactions and displayed properties similar to the Hofmeister salt series. The Hofmeister salt series has been shown to depress the LCST transition temperatures of PEGMA polymers; additionally, the hydration state of pCB polymers have been compared to Hofmeister salt series in promoting hydrophobic interactions [20,21]. pSB, pMPC and P(EG_9_)MA decreased the P(D_x_T_y_) LCSTs by ~1.3, ~2.2 and ~1.5 °C, respectively (Figure S4). Therefore, pCB was chosen as the optimal material for hydrogel fabrication as it had the greatest impact on P(D_x_T_y_) LCST.

As we have previously demonstrated that 10 wt% pCB hydrogels formed upon crosslinking copolymers of pCB-azide and pCB-DBCO that resulted in injectable, low-fouling hydrogels, we synthesized the relevant pCB-azide, pCB-DBCO copolymers and precursors (Table S1 and Figure S5) and studied their influence on P(D_x_T_y_) LCSTs [17]. We synthesized pCB-Az and pCB-DBCO copolymers with 3.5 and 3.9 mol% of azide and DBCO moieties, respectively, resulting in a hydrogel with ~3.5 mol% of crosslinks, which was previously shown to be low-fouling and enabled protein diffusion. We first confirmed that the influence of pCB on P(D_x_T_y_) LCST was concentration-dependent; 10 wt% pCB homopolymer shifted the LCST by 4.3 °C, whereas 5 wt% pCB shifted the LCST by 3.2 °C (Figure 2f, Figure S6). pCB copolymers were synthesized by polymerizing CB and APMA monomers to yield a copolymer with ~3.5 mol% APMA (Table S1). pCB-APMA was either modified with NHS-azide or NHS-DBCO to yield corresponding copolymers for hydrogel crosslinking (Figure S5). P(D_x_T_y_) LCSTs were then screened in the presence of hydrogel components and compared to a homopolymer of pCB (Figure 2h,i, Figure S7). At 5 wt%, pCB-APMA and pCB-Az decreased P(D_x_T_y_) LCSTs by an average of 2.6 and 1.8 °C, respectively, whereas pCB-DBCO increased the LCST by 1 °C due to the hydrophobicity of the DBCO moiety. The pCB hydrogel decreased the LCST of P(D_x_T_y_) by 1.7 °C, on average. Therefore, the synthesized pCB hydrogels can decrease the LCST of P(D_x_T_y_) polymers.

Ideally the P(D_x_T_y_) copolymers for the antibody conjugate would demonstrate phase transitions below 37 °C within the hydrogel and above 37 °C outside of the hydrogel for the sustained release of fully solubilized antibody drug conjugates. The D:T copolymer ratio greatly influenced LCST temperatures when different modulating polymers were added (Figure S8). Consequently, a P(D_x_T_y_) copolymer with correct D:T ratio was chosen. Only the copolymers with a D:T ratio of 30:70, P(D_30_T_70_), clearly met this criterion with LCSTs (midpoint of turbidity curve) of 36 °C and 39 °C in the hydrogel and PBS, respectively, and was therefore selected for further study. It should be noted that the LCST turbidity curve of P(D_x_T_y_) within hydrogels were broadened compared to uncrosslinked polymers. The homopolymer of triethylene glycol methyl ether methacrylate, P(T_100_), was also selected for comparison with P(D_30_T_70_) as its LCST within and outside of the hydrogel is >37 °C (Figure S7, Table S2). Additionally, both polymers showed no cytotoxicity against human lung fibroblasts (Figure S9), as expected. Therefore, they can be used and safely conjugated to therapeutic proteins.

### 3.2. Synthesis and Characterization of PEGMA-Antibody Conjugates

**Figure 3:**
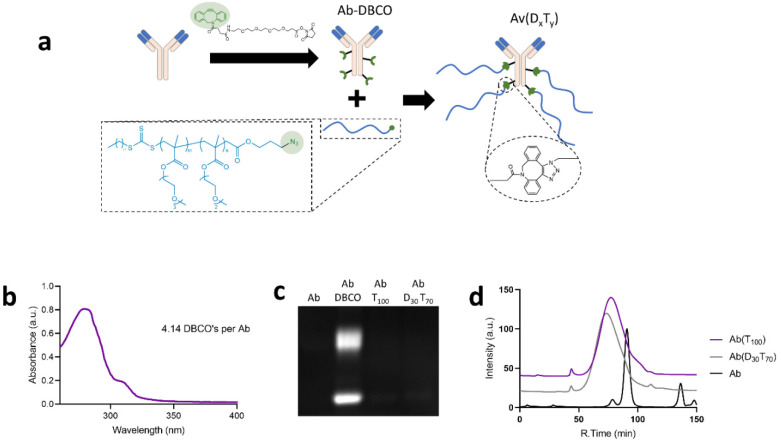
Synthesis and characterization of the Ab(D_x_T_y_) conjugates. (**a**) Illustration of Ab(D_x_T_y_) conjugate synthesis by grafting P(D_x_T_y_) to Ab-DBCO. (**b**) Absorbance spectrum of Ab-DBCO for quantification of DBCO grafting degree. (**c**) Fluorescent SDS PAGE of antibody conjugates when reacted with Cy5-azide to detect reactive DBCO groups on the antibody. Loss of fluorescence in conjugates indicates complete consumption of DBCO (Fluorescent signal indicates free DBCO reacting with Cy5-azide). (**d**) Size exclusion chromatograms of antibody-polymer conjugates.

Avastin, an anti-VEGF humanized antibody, was selected as a model antibody because it is of interest for controlled delivery applications to the posterior segment of the eye and cancerous tumors. Avastin was first modified with DBCO-PEG_4_-NHS, which resulted in an average of 4.1 DBCOs per antibody according to absorbance measurements [22]. Azide terminated P(D_x_T_y_) polymers were then grafted onto the antibody until complete consumption of antibody DBCOs according to SDS-PAGE with a fluorescent azide tag (Figure 3a–c). The antibody conjugates with P(D_30_T_70_) and P(T_100_), AbP(D_30_T_70_) and AbP(T_100_), therefore had an average of 4.1 polymers per antibody. The conjugates were then purified and characterized by size-exclusion chromatography (Figure 3d). The conjugates had short retention times and broader peaks than unmodified antibody, indicating successful polymer grafting.

**Figure 4:**
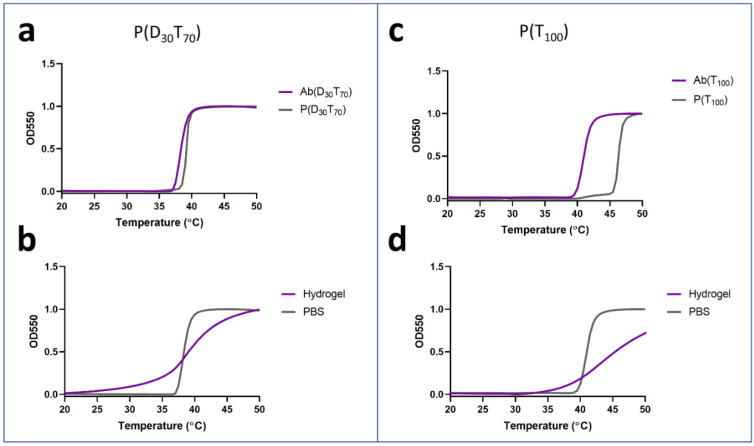
Temperature-sensitive turbidity curves of P(D_x_T_y_) copolymers (5 mg mL^−1^), Ab(D_x_T_y_) (0.5 mg/mL^−1^) in PBS and Ab(D_x_T_y_) within the pCB hydrogel. (**a**) The copolymer P(D_30_T_70_) showed similar temperature responses as a free polymer and a conjugate, Ab(D_30_T_70_). (**b**) Compared the PBS, the phase transition profile of Ab(D_30_T_70_) was broadened when encapsulated within the pCB hydrogel. (**c**) The Ab(T_100_) conjugate phase transitioned at lower temperatures than the corresponding free polymer, P(T_100_). (**d**) Compared the PBS, the phase transition profile of Ab(T_100_) was broadened when encapsulated within the pCB hydrogel.

The temperature dependence of P(D_30_T_70_) and P(T_100_) as free polymer and the conjugates, AbP(D_30_T_70_) and AbP(T_100_), were then studied in PBS and the hydrogel by following temperature-dependent phase transition with OD550 turbidity measurements (Figure 4, Figure S10), polymer concentration in all samples were held at 0.5 mg mL^−1^. Grafting polymers to the antibody shifted the turbidity curves lower with a larger effect on P(T_100_)’s LCST than P(D_30_T_70_). Compared to free polymers, the LCST of AbP(D_30_T_70_) and AbP(T_100_) shifted by 1 °C and 6 °C at 50% turbidity, respectively (Figure 4a,b). Interestingly, grafting P(T_100_) to the antibody influenced the LCST to a greater extent than P(D_30_T_70_). It has also been previously observed that grafting different PEGMA polymers to proteins had variable LCST shifts [8,23]. Encapsulating the conjugates in the hydrogel significantly broadened their phase transition curves, where even the turbidity curve begins to increase below 37 °C for the P(T_100_) conjugate. There is a similar effect (but to a lesser extent) seen from free P(D_30_T_70_) and P(T_100_) polymers in the pCB hydrogel. We hypothesize that this effect may be due to molecular crowding promoted by hydrogels, which can therefore induce phase transitions of stimuli responsive polymers at a lower temperature [24,25,26,27]. Furthermore, PEGMA phase transitions through multistep aggregation; therefore, the simultaneous effects of macromolecular crowding combined with reduced diffusivity, which influences aggregates size, can result in the broadening of phase transition temperatures curves [28,29,30]. Therefore, both the P(D_30_T_70_) and P(T_100_) conjugates can be used for controlled release applications within the pCB hydrogel. As the LCST of the conjugates are above 37 °C outside of the hydrogel, any released conjugates will be completely solubilized, which is beneficial for therapeutic applications.

### 3.3. Controlled Release of PEGMA-Protein Conjugates from pCB Hydrogels

**Figure 5:**
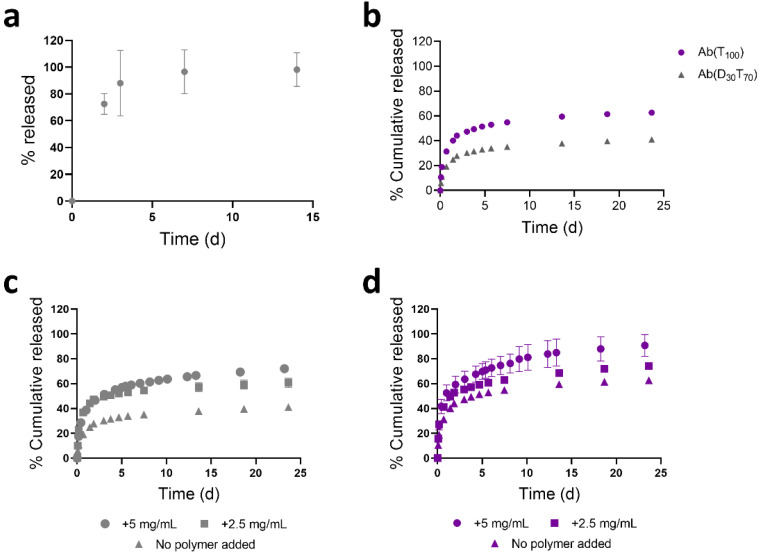
Controlled release of Ab(D_x_T_y_) release from pCB hydrogels influenced by the type of thermoresponsive polymers. (**a**) Release of the Ab(D_30_T_70_) at 4 °C demonstrating that complete release occurs within ~3 days if the conjugate is completely solubilized. (**b**) Release curves for Ab(D_30_T_70_) and Ab(T_100_) at 37 °C demonstrating that P(D_30_T_70_) lower LCST results in slower release. Release of (**c**) Ab(D_30_T_70_) and (**d**) Ab(T_100_) at 37 °C in the presence of 2.5 and 5 mgmL^−1^ of free P(D_30_T_70_) or P(T_100_), respectively, demonstrating that release is due to conjugate phase transitions (*n* = 3, ± standard deviation).

To demonstrate that controlled release is temperature-sensitive due to P(D_x_T_y_) phase transitions and not solely diffusion limited, we first investigated the release of the conjugate with the lowest LCST, Ab(D_30_T_70_), from pCB hydrogels at 4 °C, which prevents aggregation (Figure 5a). As expected, complete release of the conjugate occurred after three to seven days, indicating that the conjugate can diffuse through the hydrogel if completely solubilized.

At body temperature (37 °C), the release rate of the conjugates from the hydrogel agrees with LCST values, where partial phase transitions at lower temperatures resulted in slower released rates (Figure 5b). At three days, the conjugates differed in release by 17.7% where Ab(D_30_T_70_) and Ab(T_100_) released 31.6% and 49.3% of the initial loading. After three days, Ab(D_30_T_70_) and Ab(T_100_) sustained release rates of 73.2 ± 5.0 and 106.0 ± 8.6 ng per day, respectively (Figure S11). To further demonstrate that controlled release is due to polymer phase transition and aggregation, we conducted release experiments in the presence of free polymer corresponding to the conjugate (Figure 5c,d, Figure S11). Addition of free polymer will increase the amount of solubilized conjugate for release, which was observed in a concentration-dependent manner for both conjugates. Although the encapsulation of thermoresponsive polymer-protein conjugates in pCB hydrogels resulted in controlled delivery, the release rates plateaued at longer timepoints before complete release was achieved. Most of the difference in release profiles between Ab(D_30_T_70_) and Ab(T_100_) occurred during the first few hours and days, in a similar manner to many hydrogel affinity release systems [31,32].

**Figure 6:**
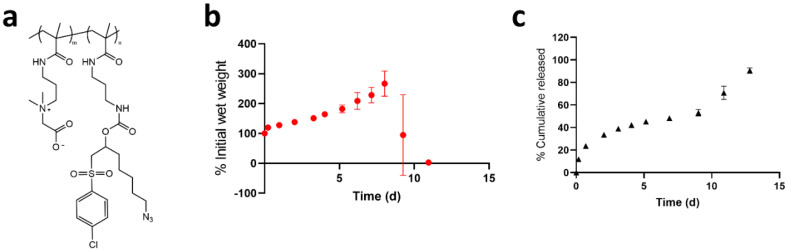
Release of Ab(D_30_T_70_) from degradable pCB hydrogels. (**a**) Structure of pCB-AzCl copolymer with hydrolytic carbamate bond for hydrolysis of hydrogel crosslinks. (**b**) Degradation of pCB hydrogels over time by following the wet weight of hydrogels gelled in microcentrifuge tubes. (**c**) Complete and sustained release of Ab(D_30_T_70_) from degradable pCB hydrogels where release is governed by the hydrogel promoted aggregation of Ab(D_30_T_70_) and hydrogel degradation (*n* = 3, ± standard deviation).

To achieve sustained and complete release of temperature-sensitive Ab(D_x_T_y_) conjugates from pCB hydrogels, we combined the hydrogel promoted polymer aggregation technology with controlled hydrogel degradation. Hydrogel degradation will promote the solubilization of the conjugate for greater release rates and eventual complete release. The pCB-Az copolymer for crosslinking with pCB-DBCO was modified to contain β eliminative carbamate bonds with reported half-lives of 36 h (Figure 6a); the hydrogel crosslinks will therefore hydrolyze for complete hydrogel degradation [15,16,33]. To demonstrate degradability, the hydrogel was formed in a pre-weighed microcentrifuge tube and wet weight was followed over time (Figure 6b); wet weight increases at first due to hydrogel swelling. The Ab(D_30_T_70_) conjugate was then encapsulated in the degradable pCB hydrogel and release was followed over 2 weeks. At first, release was similar to Ab(D_30_T_70_) in a non-degradable gel, but release increased by day 5 with a substantial increase by day 7 due to hydrogel degradation (Figure 6c). Due to the differences in hydrogel volume and surface area between the degradation study (Figure 6b) and conjugate release (Figure 6c), hydrogel degradation occurred over a longer period for the release experiment (11 vs. 13 days). Therefore, the combination of temperature-sensitive conjugate with degradable hydrogels results in sustained and complete conjugate release.

## 4. Conclusions

Polymer-protein therapeutics, including PEGMA conjugates, continue to represent promising new therapeutics that may benefit from sustained release vehicles [2]. Here, we demonstrated that a zwitterionic hydrogel, pCB, can locally depress the LCST of thermoresponsive PEGMA-protein conjugates for their sustained release as solubilized molecules. In combination with a degradable pCB hydrogel, complete and sustained release of the conjugate occurred over 13 days. Therefore, the design of thermoresponsive polymers and hydrogels can yield drug delivery systems where polymer-protein conjugates aggregate within but not outside of hydrogels, representing a new mechanism for the controlled delivery of polymer conjugates.

## Data Availability

The data presented in this study are available on request from the corresponding author.

## Supplementary Materials

The following are available online at https://www.mdpi.com/article/10.3390/polym13162772/s1, Figure S1: ^1^H NMR of P(D_x_T_y_) in D_2_O synthesized through RAFT polymerization, Figure S2: ^1^H NRM spectra and chemical structures of the modulating polymers used, Figure S3: Temperature turbidity curves (OD550) of P(D_x_T_y_) (5 mg mL^−1^) with modulating polymers (50 mg mL^−1^) in PBS, Figure S4: Decrease in heating and cooling LCST transition temperatures of P(D_x_T_y_) polymers with he addition of zwitterionic polymer, Figure S5 Chemical structures of (a) pCB-APMA, (b) pCB-DBCO, (c) pCB-Az and their respective ^1^H NMR spectra in D_2_O (d), Figure S6: Temperature turbidity curves (OD550) of PEGMA polymers with pCB at different concentrations in PBS, Figure S7: Temperature turbidity curves (OD550) of PEGMA polymers with hydrogel precursor polymers and encapsulated within pCB hydrogel in PBS, Figure S8: Phase transition temperatures of PEGMA polymers when heating and cooling plotted with respect to triethylene glycol content with he addition of modulating polymers, different pCB concentrations and hydrogel precursors. Slopes of the aggregated data, Figure S9: Cytotoxicity of P(T_100_) and P(D_30_T_70_) polymers incubated with 10,000 cells of human lung fibroblasts compared to PBS controls. No cytotoxicity from the polymers was observed, Figure S10: Temperature turbidity curves of protein polymer conjugates and their corresponding polymer in PBS, Figure S11: First order curves of release after day 3 of Ab(D_30_T_70_) and Ab(T_100_), Table S1: MW of polymers synthesized obtained from GPC with PEG standards, Table S2: Phase transition temperatures of PEGMA polymers with the addition of modulating polymers and hydrogel precursor polymers when heating and cooling.
