# Supplementary material for: Modulating the Thermoresponse of Polymer-Protein Conjugates with Hydrogels for Controlled Release
Source: Polymers (Basel). 2021 Aug 18;13(16):2772. doi: 10.3390/polym13162772 (PMC8399950; doi:10.3390/polym13162772)
Supplement: Supplementary file 1 [file polymers-13-02772-s001.zip › polymers-1313081-supplementary.pdf]

Supplementary Material

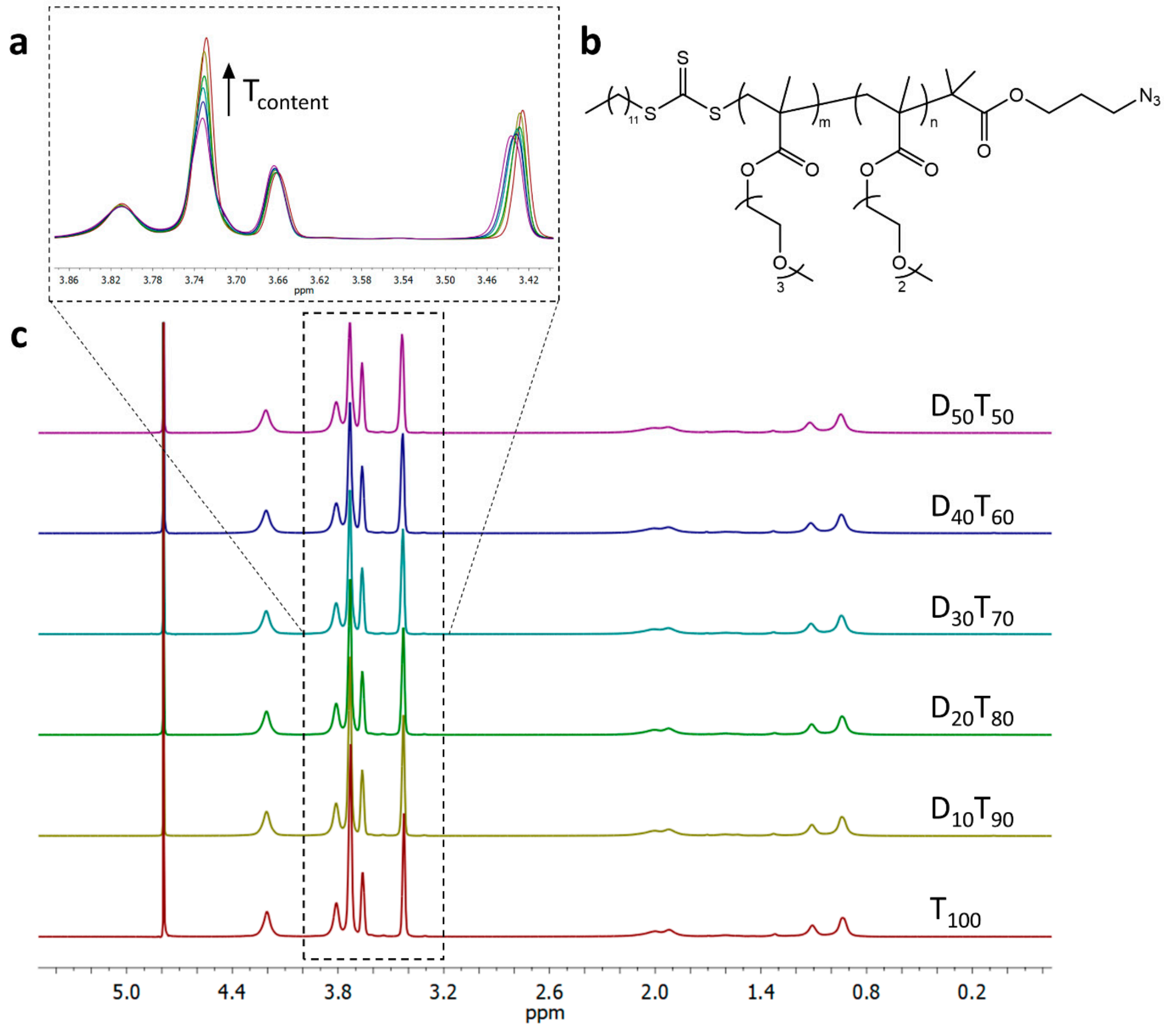

Figure S1. ^1H NMR of $P(D_xT_y)$ in D_2O synthesized through RAFT polymerization. (a) Increase in peak intensity correlates with increase in T monomer content. (b) Chemical structure of PEGMA polymers synthesized. (c) ^1H NMR spectra of the different $P(D_xT_y)$ polymers in the study.

Polymer	M_W	M_n	Đ	APMA mol%
T_{100}	26 362	17 098	1.54	-
$D_{10}T_{90}$	39 909	25 541	1.56	-
$D_{20}T_{80}$	31 098	20 085	1.55	-
$D_{30}T_{70}$	43 991	27 809	1.58	-
$D_{40}T_{60}$	42 379	25 963	1.63	-
$D_{50}T_{50}$	37 661	24 416	1.54	-
pCB-APMA	43 768	31 457	1.39	3.5
pCB	26 160	24 031	1.09	-
pSB	65 734	57 695	1.14	-
pMPC	34 974	31 370	1.12	-
P(EG$_9$)MA	67 210	51 810	1.30	-

Table S1. MW of polymers synthesized obtained from GPC with PEG standards.

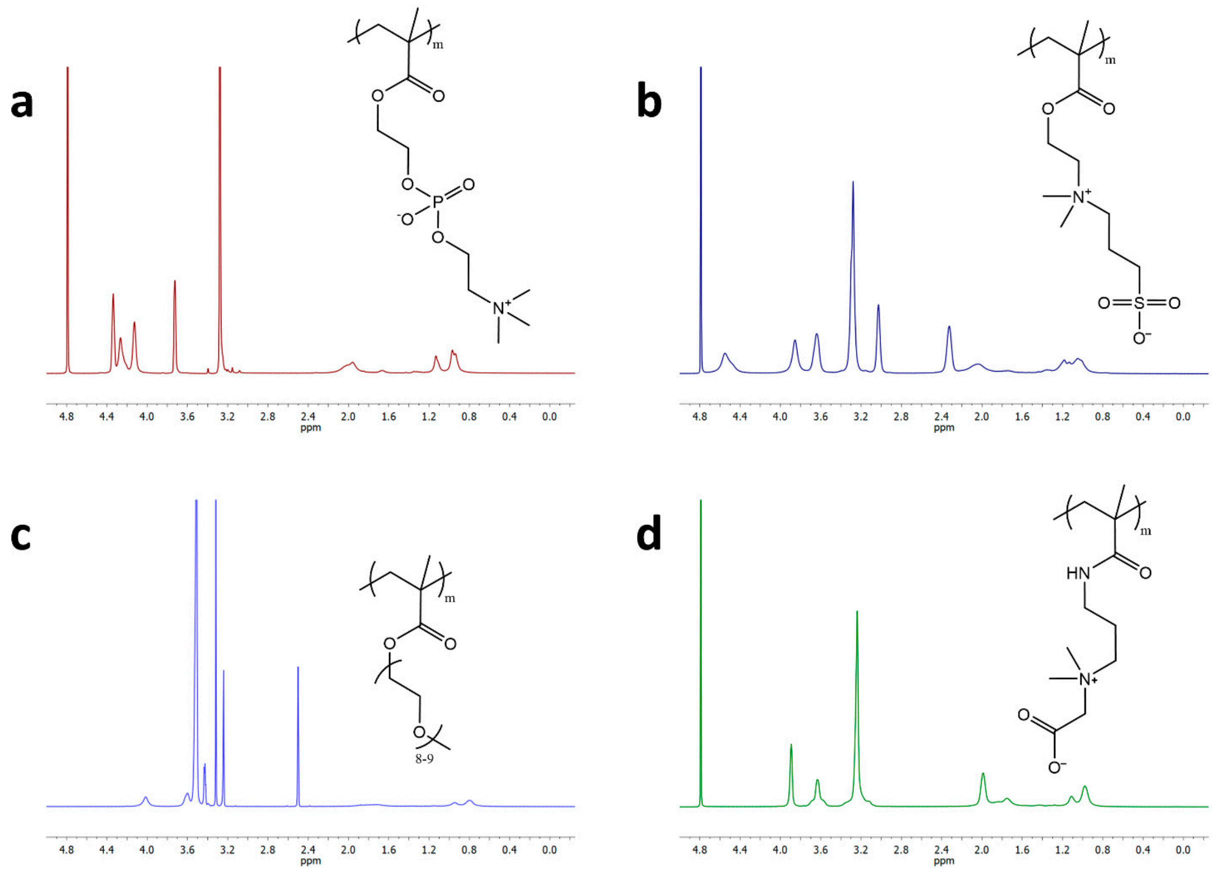

Figure S2. ^{1}H NMR spectra and chemical structures of the modulating polymers used: (a) pMPC, (b) pSB, (c) P(EG_9)MA and (d) pCB.

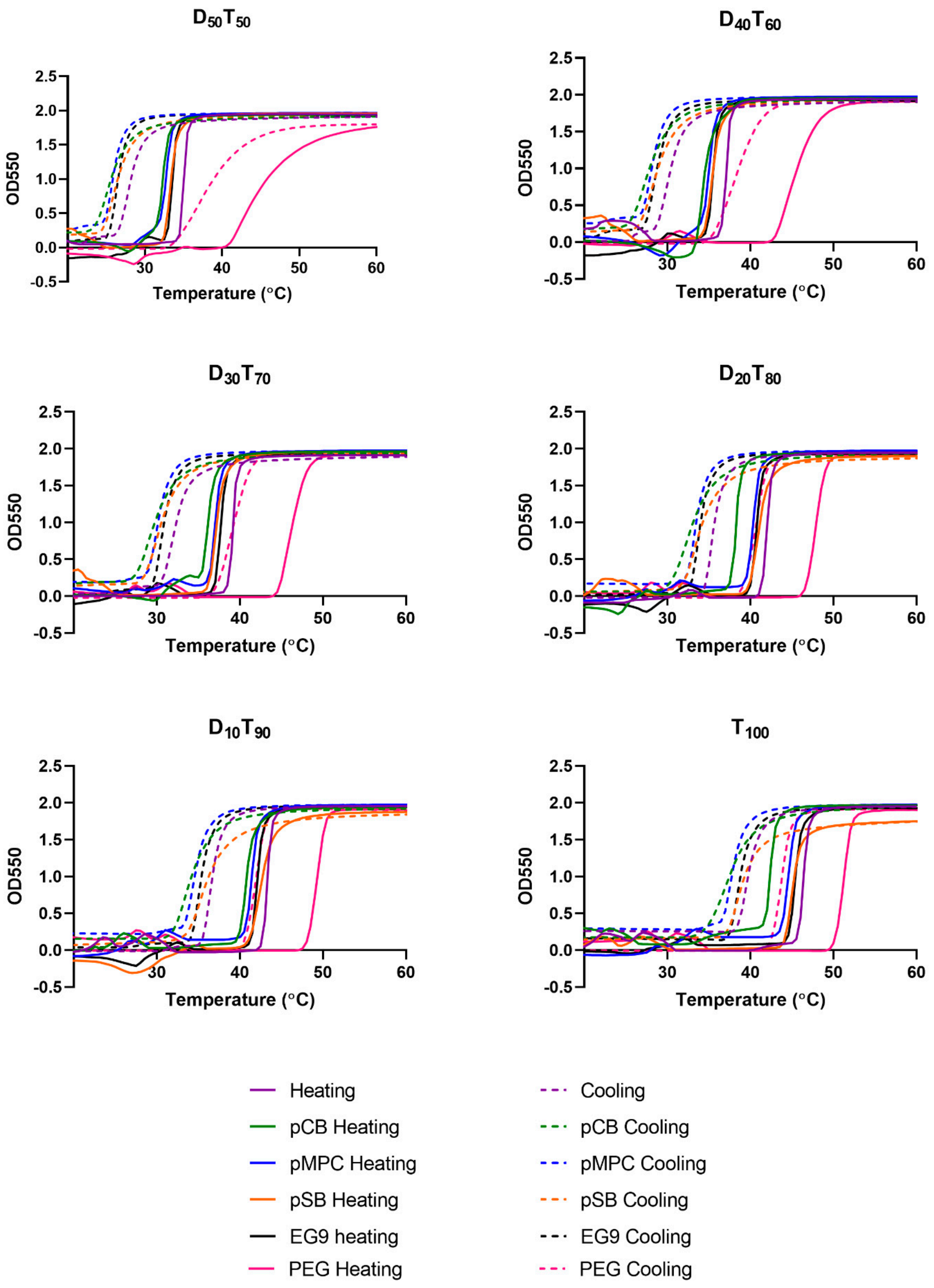

Figure S3. Temperature turbidity curves (OD550) of $P(D_xT_y)$ (5 mg mL^{-1}) with modulating polymers (50 mg mL^{-1}) in PBS.

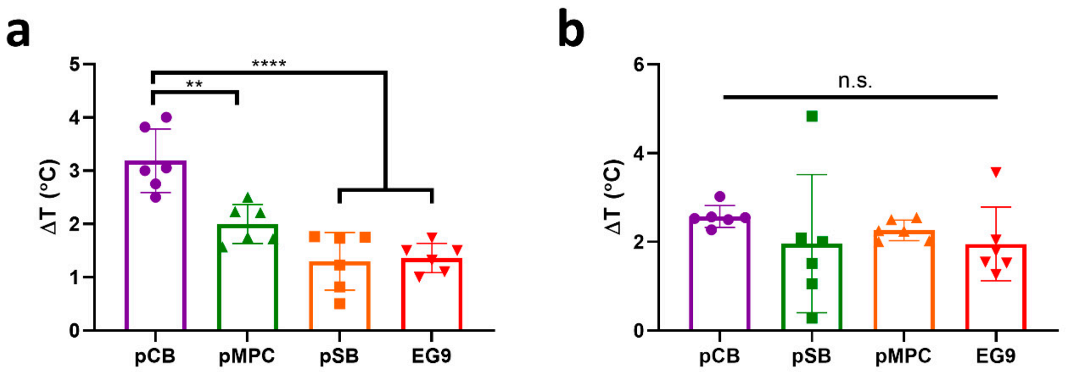

Figure S4. Decrease in (a) heating and (b) cooling LCST transition temperatures (50% of maximal signal) of $P(D_xT_y)$ polymers with the addition of zwitterionic polymer (n = 6, ± standard deviation, one way ANOVA, Tukey's post-test).

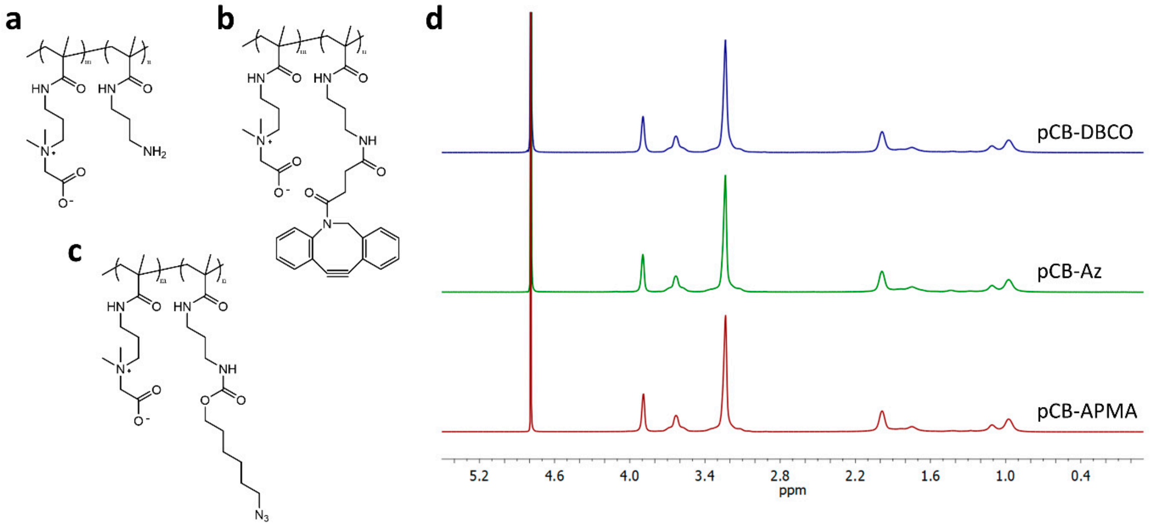

Figure S5. Chemical structures of (a) pCB-APMA, (b) pCB-DBCO, (c) pCB-Az and their respective 1-H NMR spectra in D_2O (d).

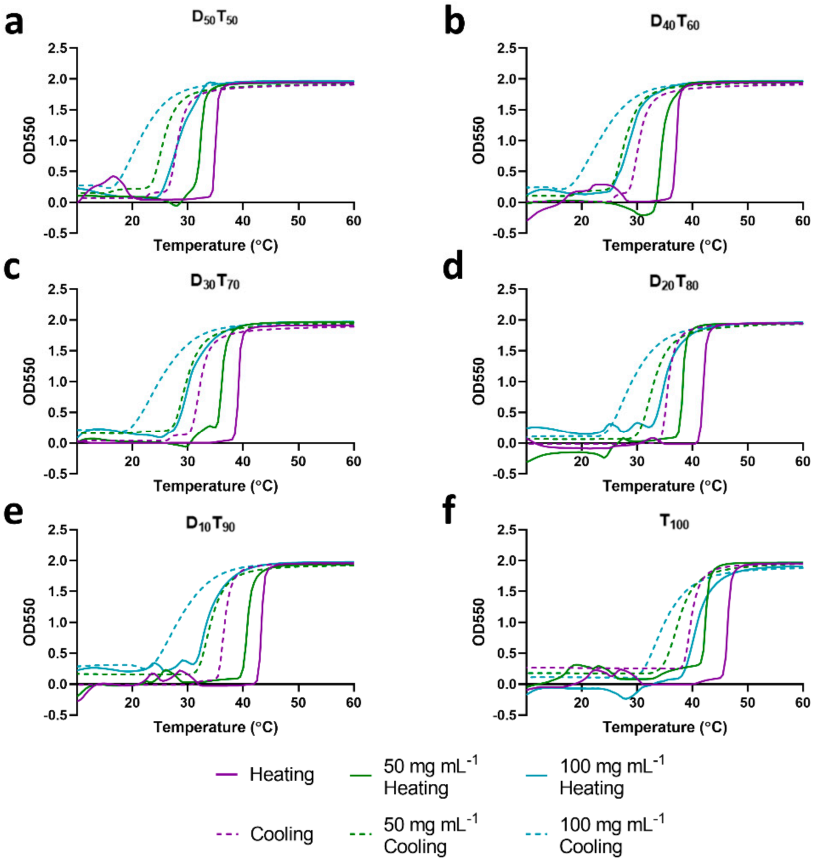

Figure S6. Temperature turbidity curves (OD550) of PEGMA polymers (5 mg mL^{-1}) with pCB with at different concentrations (50 mg mL^{-1} and 100 mg mL^{-1}) in PBS.

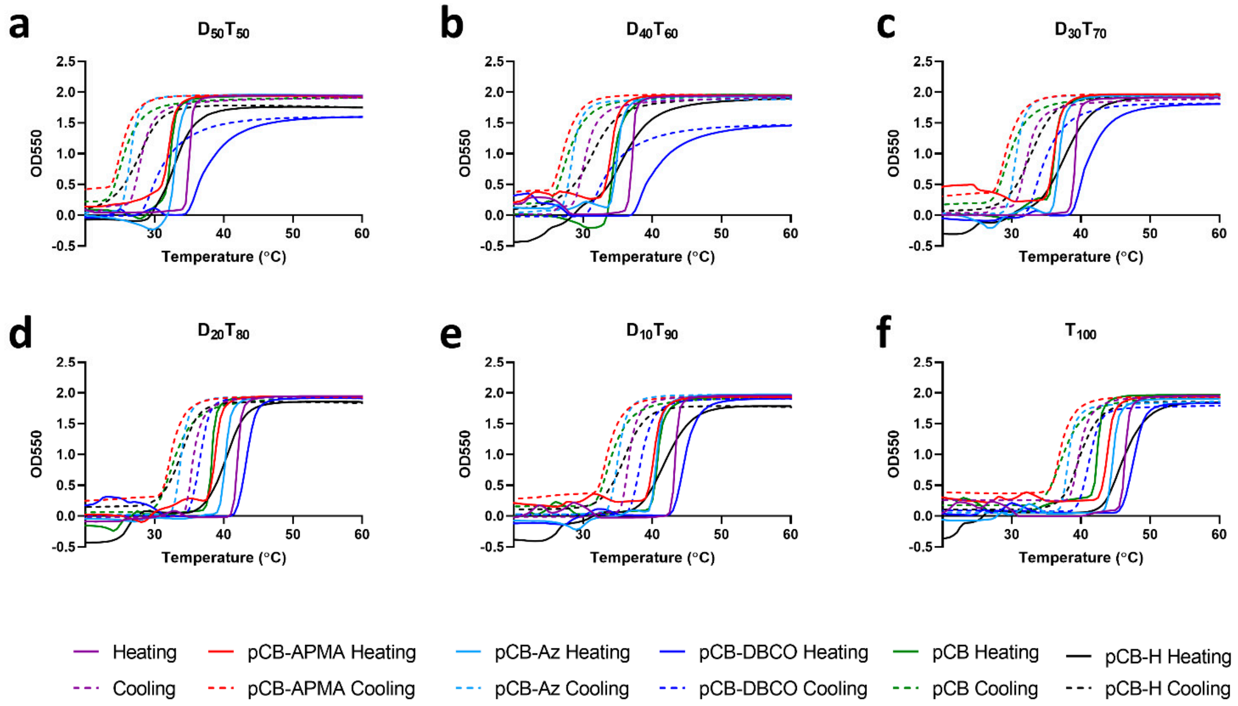

Figure S7. Temperature turbidity curves (OD550) of PEGMA polymers (5 mg mL^{-1}) with hydrogel precursor polymers (50 mg mL^{-1}) and encapsulated within a pCB hydrogel (pCB-H, 100 mg mL^{-1}) in PBS.

a

Heating	Control	pCB	pSB	pMPC	P(EG9)MA	pCB (10 wt%)	PEG	pCB-APMA	pCB-Az	pCB-DBCO	pCB-H
T_{100}	46.25	42.25	45.02	44.53	45.15	41.02	51.25	43.75	44.75	47.75	46.49
$D_{10}T_{90}$	43.25	40.75	42.75	41.50	42.25	33.50	49.25	40.25	40.75	45.02	42.52
$D_{20}T_{80}$	42.07	38.25	41.25	40.50	40.75	35.01	48.02	38.75	40.25	43.50	38.03
$D_{30}T_{70}$	39.25	36.20	37.50	37.04	37.75	30.60	46.49	36.25	37.06	41.51	36.03
$D_{40}T_{60}$	37.25	34.50	35.52	35.02	35.51	28.55	45.55	34.01	34.99	42.51	36.53
$D_{50}T_{50}$	35.25	32.25	33.49	32.75	33.75	28.51	46.02	32.06	32.25	39.02	33.54

b

Cooling	Control	pCB	pSB	pMPC	P(EG9)MA	pCB (10 wt%)	PEG	pCB-APMA	pCB-Az	pCB-DBCO	pCB-H
T_{100}	40.51	37.49	39.46	38.01	38.99	34.96	44.01	37.01	38.25	41.48	40.01
$D_{10}T_{90}$	36.75	34.25	36.47	34.50	35.49	27.99	42.01	33.51	35.25	38.45	36.51
$D_{20}T_{80}$	35.75	33.48	30.92	33.51	33.96	28.97	40.75	32.48	33.97	36.75	33.98
$D_{30}T_{70}$	32.49	29.93	30.49	30.47	28.93	24.99	39.50	29.25	30.75	35.47	33.00
$D_{40}T_{60}$	30.50	27.97	28.99	28.50	28.96	23.00	38.48	27.00	28.45	37.00	31.45
$D_{50}T_{50}$	28.50	25.95	26.41	25.95	26.46	21.46	39.01	24.97	26.75	32.49	27.90

Table S2. Phase transition temperatures (defined by 50% of maximum turbidity) of PEGMA polymers with the addition of modulating polymers and hydrogel precursor polymers when heating (a) and cooling (b).

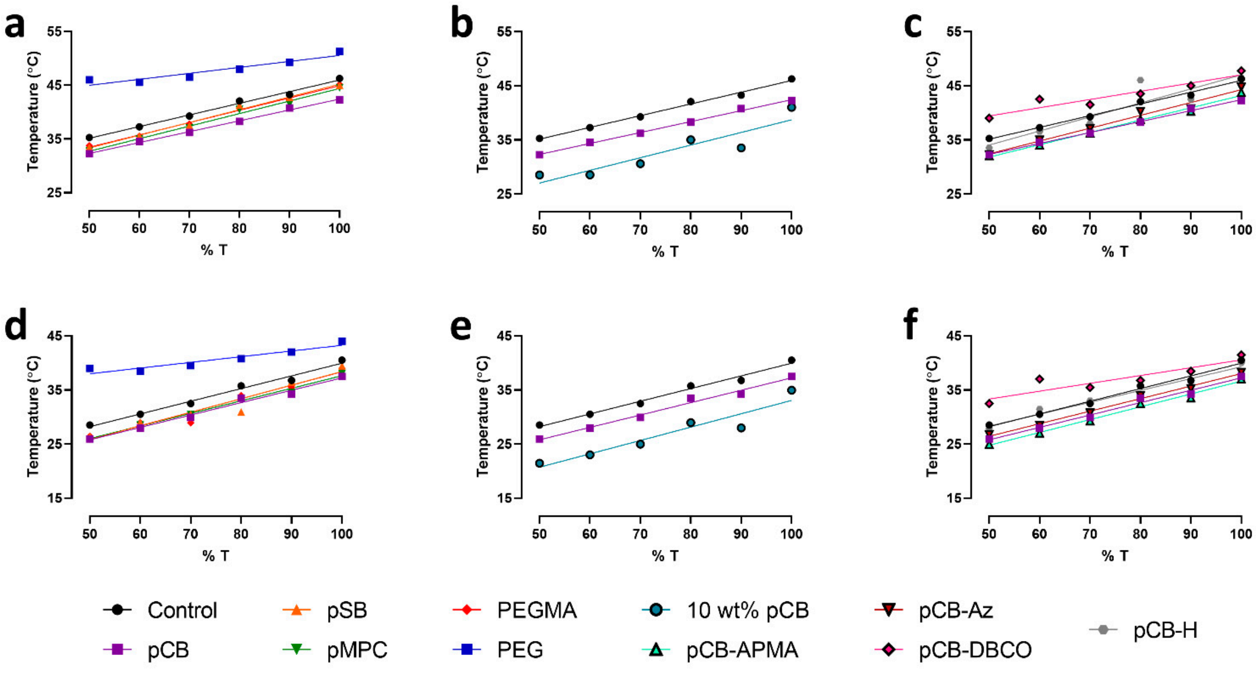

g

	Heating		Cooling	
	Slope	R^2	Slope	R^2
**Control**	0.217 ± 0.010	0.992	0.235 ± 0.015	0.984
**pCB**	0.202 ± 0.006	0.997	0.229 ± 0.014	0.984
**pSB**	0.237 ± 0.012	0.989	0.252 ± 0.035	0.930
**pMPC**	0.234 ± 0.012	0.989	0.232 ± 0.013	0.988
**P(EG9)MA**	0.229 ± 0.009	0.994	0.249 ± 0.028	0.953
**pCB (10 wt%)**	0.111 ± 0.019	0.896	0.105 ± 0.019	0.887
**PEG**	0.234 ± 0.052	0.837	0.247 ± 0.042	0.898
**pCB-APMA**	0.228 ± 0.011	0.991	0.237 ± 0.013	0.988
**pCB-Az**	0.237 ± 0.018	0.979	0.232 ± 0.011	0.991
**pCB-DBCO**	0.152 ± 0.025	0.899	0.145 ± 0.035	0.814
**pCB-H**	0.259 ± 0.058	0.835	0.219 ± 0.020	0.967

Figure S8. Phase transition temperatures of PEGMA polymers (5 mg mL^{-1}) when (a, b, c) heating and (d, e, f) cooling plotted with respect to triethylene glycol content with the addition of (a, d) modulating polymers, (b, e) different pCB concentrations and (c, f) hydrogel precursors. (g) Slopes of the aggregated data.

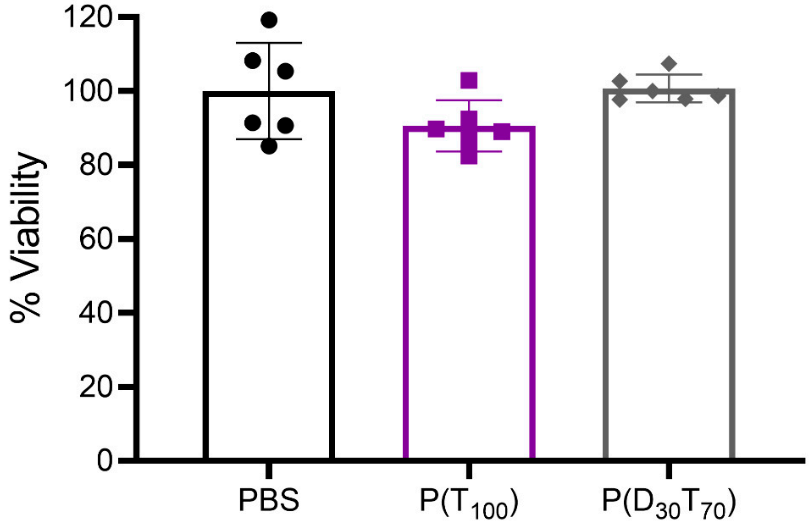

Figure S9. Cytotoxicity of P(T_{100}) and P($D_{30}T_{70}$) (1 mg mL^{-1}) polymers incubated with 10 000 cells of human lung fibroblasts (n = 6, ± standard deviation) compared to PBS controls. No cytotoxicity from the polymers was observed.

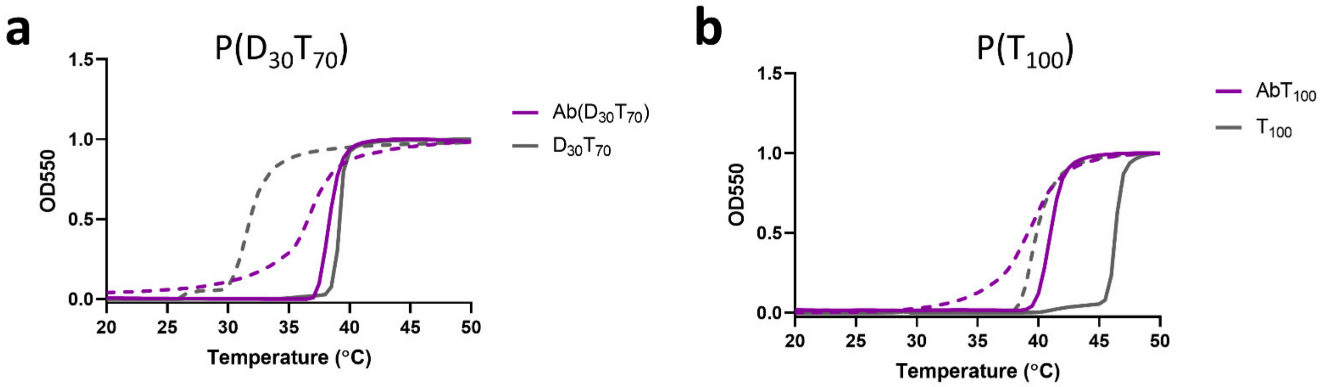

Figure S10. Temperature turbidity curves of protein polymer conjugates and their corresponding polymer in PBS. Heating is represented by the solid lines and cooling represented by the dashed lines: (a) Ab($D_{30}T_{70}$) (0.25 mg mL^{-1}) and corresponding polymer (5 mg mL^{-1}) in PBS; (b) Ab(T_{100}) (0.25 mg mL^{-1}) and corresponding polymer (5 mg mL^{-1}) in PBS.

a **b**

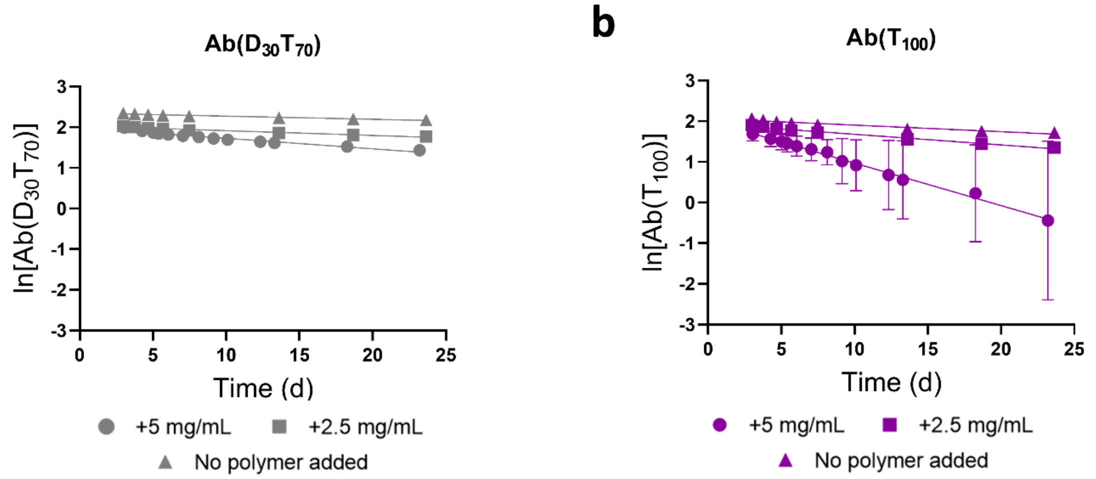

c

	+ 5 mg mL^{-1}		+ 2.5 mg mL^{-1}		No polymer added	
	k_{eff}	R^2	k_{eff}	R^2	k_{eff}	R^2
**Ab($D_{30}T_{70}$)**	2.6 x 10^{-2} ± 1.2 x 10^{-3}	0.9106	1.2 x 10^{-2} ± 1.7 x 10^{-3}	0.6892	7.7 x 10^{-3} ± 4.9 x 10^{-4}	0.9173
**Ab(T_{100})**	1.0 x 10^{-1} ± 1.9 x 10^{-2}	0.4572	2.6 x 10^{-2} ± 1.7 x 10^{-3}	0.9177	1.6 x 10^{-2} ± 1.2 x 10^{-3}	0.8923

d

	+ 5 mg mL^{-1}		+ 2.5 mg mL^{-1}		No polymer added	
	Release rate after 3 d (ng d^{-1})	R^2	Release rate after 3 d (ng d^{-1})	R^2	Release rate after 3 d (ng d^{-1})	R^2
**Ab($D_{30}T_{70}$)**	142.1 ± 9.0	0.87	76.9 ± 11.1	0.69	73.2 ± 5.0	0.91
**Ab(T_{100})**	199.5 ± 32.7	0.50	133.3 ± 9.9	0.89	106.0 ±8.6	0.87

Figure S11. First order curves of release after day 3 of (a) Ab($D_{30}T_{70}$) and (b) Ab(T_{100}). (c) k_{eff} of the first order release curves calculated through linear regression. (d) Release rate of AbD_xT_y conjugates after day 3.
